# Aggregation of Vascular Risk Factors Modulates the Amplitude of Low-Frequency Fluctuation in Mild Cognitive Impairment Patients

**DOI:** 10.3389/fnagi.2020.604246

**Published:** 2020-12-21

**Authors:** Liying Zhuang, Huafu Ni, Junyang Wang, Xiaoyan Liu, Yajie Lin, Yujie Su, Kan Zhang, Yaguo Li, Guoping Peng, Benyan Luo

**Affiliations:** ^1^Department of Neurology, First Affiliated Hospital, Zhejiang University School of Medicine, Hangzhou, China; ^2^Department of Neurology, Zhejiang Hospital, Hangzhou, China; ^3^Department of Neurology, Beilun People's Hospital, Ningbo, China

**Keywords:** mild cognitive impairment, functional magnetic resonance imaging, amplitude of low frequency fluctuation, vascular risk, cognition

## Abstract

**Background:** Several vascular risk factors, including hypertension, diabetes, body mass index, and smoking status are found to be associated with cognitive decline and the risk of Alzheimer's disease (AD). We aimed to investigate whether an aggregation of vascular risk factors modulates the amplitude of low-frequency fluctuation (ALFF) in patients with mild cognitive impairment (MCI).

**Methods:** Forty-three MCI patients and twenty-nine healthy controls (HCs) underwent resting-state functional MRI scans, and spontaneous brain activity was measured by the ALFF technique. The vascular risk profile was represented with the Framingham Heart Study general cardiovascular disease (FHS-CVD) risk score, and each group was further divided into high and low risk subgroups. Two-way ANOVA was performed to explore the main effects of diagnosis and vascular risk and their interaction on ALFF.

**Results:** The main effect of diagnosis on ALFF was found in left middle temporal gyrus (LMTG) and left superior parietal gyrus (LSPG), and the main effect of risk on ALFF was detected in left fusiform gyrus (LFFG), left precuneus (LPCUN), and left cerebellum posterior lobe (LCPL). Patients with MCI exhibited increased ALFF in the LMTG and LSPG than HCs, and participants with high vascular risk showed increased ALFF in the LFFG and LCPL, while decreased ALFF in the LPCUN. An interaction between diagnosis (MCI vs. HC) and FHS-CVD risk (high vs. low) regarding ALFF was observed in the left hippocampus (LHIP). HCs with high vascular risk showed significantly increased ALFF in the LHIP than those with low vascular risk, while MCI patients with high vascular risk showed decreased ALFF in the LHIP than HCs with high vascular risk. Interestingly, the mean ALFF of LHIP positively correlated with word recall test in HCs with high vascular risk (rho = 0.630, *P* = 0.016), while negatively correlated with the same test in MCI patients with high vascular risk (rho = −0.607, *P* = 0.001).

**Conclusions:** This study provides preliminary evidence highlighting that the aggregation of vascular risk factors modulates the spontaneous brain activity in MCI patients, and this may serve as a potential imaging mechanism underlying vascular contribution to AD.

## Introduction

Alzheimer's disease (AD) is a cognitive continuous ranging from cognitively unimpaired, mild cognitive impairment (MCI) to dementia (McKhann et al., 2011; Dubois et al., 2014). Genetic susceptibility as well as environmental factors and their interaction over the life span contributes to a variety of pathological process in the clinical expression of dementia (Qiu et al., 2009). Several vascular risk factors such as hypertension, diabetes, dyslipidemia, and obesity appear to increase AD risk, which suggests the contribution of vascular factors to the pathogenesis of this condition (Breteler, 2000; Purnell et al., 2009). Previous clinical trials have demonstrated that about thirty percent of AD cases are attributed to seven controllable risk factors, including high blood pressure, diabetes, obesity, smoking, depression, low level of physical activity, and education (Barnes and Yaffe, 2011; Norton et al., 2014). Some studies have evaluated the effect of treatment of a single vascular risk factor on cognitive deterioration, however, results were controversial (Launer et al., 2000; Tan et al., 2003; Ninomiya et al., 2011; Safouris et al., 2015). The aggregative effects of vascular risk factors may better illustrate the role of vascular components in cognitive deterioration.

The Framingham Heart Study general cardiovascular disease (FHS-CVD) risk profile is a globally, well-validated and multivariable assessment of overall cardiovascular risk (D'Agostino et al., 2008). The FHS-CVD risk is quantified by weighting sex, age, systolic blood pressure (SBP), treatment for hypertension, current smoking status, diabetes, and body mass index (BMI) and predicts the risk of vascular events over a 10-year period. Furthermore, the FHS-CVD risk can represent the influence of vascular risk factors on structural and functional changes of macro- and micro-vessels. The FHS-CVD risk score can also predict the progression of cognitive decline in AD and the risk of progression from MCI to dementia (Viticchi et al., 2015, 2017). Moreover, vascular risk represented by FHS-CVD risk acts alone and synergistically with β-amyloid (Aβ) pathology to promote cognitive decline in cognitively normal elderly adults (Rabin et al., 2018). While there has been extensive evidence showing the effects of vascular risk factors on disease, the mechanisms of vascular involvement in cognitive impairment, as well as dementia and AD, remain not fully understood.

Functional neuroimaging techniques sensitive to spatial patterns of blood oxygenation or blood flow are important tools for investigating the functional organization of the human brain. Functional MRI (fMRI) is a good example of this field. In 1995, it has been demonstrated that blood oxygen level-dependent (BOLD) low frequency oscillations (<0.1 Hz) in resting-state fMRI were of physiological significance and were closely associated with brain spontaneous neuronal activity (Biswal et al., 1995). He et al. firstly introduced amplitude of low-frequency fluctuation (ALFF) to quantitatively measure regional BOLD signal variation in attention deficit hyperactivity disorder (He et al., 2007). To date, ALFF has been proved to be a reliable and useful indicator to characterize the spontaneous neuronal activity of the brain in MCI or AD patients (Wang et al., 2011; Cha et al., 2015). The purpose of our study was to explore whether the aggregation of vascular risk factors represented by FHS-CVD risk modulates the spontaneous brain activity in MCI patients.

## Materials and Methods

### Participants and Clinical Evaluation

In this study, 43 MCI subjects and 29 healthy controls (HCs) were enrolled from the Memory Clinic of the First Affiliated Hospital, Zhejiang University School of Medicine. Written informed consent were collected from all the subjects prior to participating in the study, in accordance with protocols approved by the Ethics Committee of the First Affiliated Hospital of Zhejiang University (reference number: 2016-315).

Demographic data and medical history were collected from all of the participants. Each of them underwent general and neurological examination and a comprehensive neuropsychological battery assessment, including Clinical Dementia Rating (CDR), Mini-Mental State Examination (MMSE), Montreal Cognitive Assessment (MoCA), and the 14-item AD Assessment Scale-Cognitive (ADAS-Cog) subscale.

### Inclusion and Exclusion Criteria

MCI patients met the following criteria (Winblad et al., 2004): (1) 54 to 80 years old; (2) years of education ≥ 6; (3) a subjective complaint of memory; (4) an objective memory impairment <1.5 SD for age adjusted and education adjusted norms; (5) CDR = 0.5; (6) MMSE score was 20 or higher for subjects with 6 years and 24 or higher for those with 9 years of education; (7) normal activities of daily living; (8) not demented. In addition, HCs met the following conditions: CDR = 0; MMSE score of 20 or higher for subjects with 6 years and 24 or higher for subjects with 9 years of education (Katzman et al., 1988).

Subjects with prior stroke (Hachinski ischemic score >4), traumatic brain injury, Parkinson's disease, epilepsy, alcoholism, major depression or other neuropsychiatric conditions, or severe visual or hearing loss were all excluded from this study.

### FHS-CVD risk

For each participant, the FHS-CVD risk score was quantified by the calculator provided by the American Heart Association and the American College of Cardiology (https://framinghamheartstudy.org/fhs-risk-functions/cardiovascular-disease-10-year-risk/). The information required for FHS-CVD risk includes age, sex, SBP, antihypertensive treatment, diabetes, smoking, and BMI (D'Agostino et al., 2008). The FHS-CVD risk provides a 10-year risk prediction of future cardiovascular events (defined as coronary death, non-fatal myocardial infarction, angina, heart failure, fatal or non-fatal stroke, transient ischemic attack, and peripheral artery disease). Subjects were clustered into two subgroups based on FHS-CVD risk scores (low: <10%, high: ≥10%) as in a previous study (Hou et al., 2018), scince guidelines from the American Heart Association recommend aspirin for patients with a 10-year risk more than 10% (Pearson et al., 2002).

### MRI Scanning

All subjects were scanned using a General Electric 3.0 Tesla scanner (General Electric Medical Systems, Waukesha, WI, United States) with a standard head coil. Functional images were obtained by a gradient-recalled echo-planar imaging (GRE-EPI) sequence: repetition time (TR) = 2,000 ms; echo time (TE) = 30 ms; flip angle (FA) = 90°; acquisition matrix = 64 × 64; field of view (FOV) = 220 × 220 mm^2^; thickness = 3.2 mm; gap = 0 mm; number of slices = 43. T1-weighted anatomical images were acquired by a 3D-magnetization prepared rapid gradient-echo (MPRAGE) sequence: TR = 8.2 ms; TE = 3.18 ms; FA = 8°; acquisition matrix = 256 × 256; FOV = 256 × 256 mm^2^; thickness = 1.0 mm; gap = 0 mm; number of slices = 176. During the scan, subjects were asked to keep their eyes closed, remain still, and not to fall asleep.

### Imaging Pre-processing

Imaging preprocessing were performed with SPM12 (http://www.fil.ion.ucl.ac.uk/spm) and RESTplus V1.2 (http://www.restfmri.net/forum/RESTplusV1.2). The steps of imaging preprocessing were the same as described previously (Zhuang et al., 2019). The first 10 volumes were discarded for the participants' adaption to the scanner. Timing differences and head-motion effects of the remaining volumes were further corrected. Participants with head motion of translation or rotation parameters exceeding ± 3 mm or ± 3° were excluded. The head motion index estimated by a realignment algorithm was calculated for each participant, and the difference between groups was statistically analyzed. Next, the T1-weighted structural images were co-registered to the motion corrected mean functional images using a linear transformation. Gray matter, white matter and cerebrospinal fluid were further segmented for the transformed structural images with a unified segmentation algorithm (Ashburner and Friston, 2005). The corrected functional images were normalized to the Montreal Neurological Institute (MNI) space and re-sampled to a voxel size of 3 × 3 × 3 mm^3^ voxels. The resulting images were spatially smoothed with the full width at half maximum (FWHM) of 6 mm. The last step was the removal of linear trend of time courses.

### Calculation of ALFF

ALFF values were calculated using the RESTplus software as in previous studies (He et al., 2007; Zhuang et al., 2019). After data preprocessing, the time series were transformed to the frequency domain of 0.01~0.08 Hz and the power spectrum was obtained for each given voxel. Secondly, the square root of the power spectrum obtained with the Fast Fourier Transform was averaged between 0.01 and 0.08 Hz. This averaged square root was considered as ALFF. Finally, standardization was performed by dividing the ALFF value of each voxel with the global mean ALFF value within a whole-brain mask.

### Statistical Analysis

Two-way analysis of variance (ANOVA) was carried out for the comparison of continuous demographic and neuropsychological data, and chi-square test was applied for categorical variables. The main effects of diagnosis (MCI vs. HC) and FHS-CVD risk (high vs. low), and the interaction between diagnosis and risk were analyzed. All of the statistical analyses above were performed by SPSS 17.0, with *P* < 0.05 as statistically significant.

A voxel-wise ANOVA was performed to analyze the main effects of diagnosis, the main effects of FHS-CVD risk, and the interactions between diagnosis and FHS-CVD risk on ALFF maps using SPM12 (http://www.fil.ion.ucl.ac.uk/spm). The significant clusters of the main effects and interactions were further analyzed by *post-hoc* tests. Multiple comparison correction was carried out for the statistical maps, based on Gaussian random field theory (GRF) (voxel-wise *P* < 0.01, cluster-wise *P* < 0.05, two-tailed) as in previous studies (Yang et al., 2019; Zhou et al., 2019; Huang et al., 2020). At last, we performed a correlative analysis between the ALFF values of the significant clusters of interactions and the neuropsychological test scores (*P* < 0.05).

## Results

### Demographics and Clinical Data

The excessive motion artifacts or distortions of the images were visually inspected to ensure adequate quality. The head motion index (framewise displacement, FD) was calculated for each participant (Power et al., 2013). The average FD values were compared across different subgroups using two-way ANOVA. Neither main effect of diagnosis (*F* = 3.032, *P* = 0.086), vascular risk (*F* = 0.018, *P* = 0.893), nor interaction between diagnosis and vascular risk (*F* = 0.713, *P* = 0.401) was found on FD.

The demographics profiles of MCI patients and HCs stratified by FHS-CVD risk were shown in Table 1. The demographics of MCI patients and HCs, including age, education years, gender ratio were all matched. There were no significant differences of each vascular risk factor and FHS-CVD risk between MCI patients and HCs. Subjects with high FHS-CVD risk got older age, more males, more diabetic patients, and more hypertensive patients with higher SBP. While, there were no significant differences of education years between different FHS-CVD risk subgroups in either MCI or HC groups. Compared with controls, MCI patients exhibited deficits as noted on MMSE, MoCA, word recall test, following commands, delayed word recall, and ADAS-Cog 14. There was no significant difference of neuropsychological test score except for MMSE and Construction scores between high and low FHS-CVD risk. We did not find any interaction between diagnosis and FHS-CVD risk on neuropsychological data (for details see Supplementary Table 1).

**Table 1:** Demographics profiles of MCI patients and HCs stratified by FHS-CVD risk.

	**MCI group (*****N*** **= 43)**		**HC group (*****N*** **= 29)**		***P***		
	**Low risk (*N* = 17)**	**High risk (*N* = 26)**	**Low risk (*N* = 15)**	**High risk (*N* = 14)**	**diagnosis**	**risk**	**interaction**
Age (years)	60.71 ± 6.32	64.50 ± 5.64	58.20 ± 4.92	66.79 ± 3.68	0.933	<0.001^*^	0.070
Education (years)	9.35 ± 2.80	9.73 ± 3.27	10.40 ± 3.44	11.43 ± 2.79	0.074	0.355	0.668
Gender (male/female)	0/17	18/8	1/14	6/8	0.291	<0.001^*^	0.079
SBP (mmHg)	117.47 ± 7.75	125.58 ± 15.52	116.93 ± 10.84	126.21 ± 13.74	0.987	0.007^*^	0.850
BMI (Kg/m^2^)	22.73 ± 3.12	24.10 ± 2.77	22.46 ± 1.78	23.94 ± 4.19	0.770	0.055	0.938
Antihypertension (yes/no)	0/17	11/15	0/15	8/6	0.423	<0.001^*^	0.423
Diabetes (yes/no)	0/17	5/21	0/15	3/11	0.883	0.008^*^	0.883
Current smoker (yes/no)	0/17	6/20	0/15	0/14	0.072	0.072	0.072
FHS-CVD risk	0.06 ± 0.02	0.24 ± 0.09	0.06 ± 0.02	0.20 ± 0.08	0.180	<0.001^*^	0.312

*Values were described as the mean ± standard deviation (SD). The P-values were obtained by two-way analysis of variance (ANOVA)*.

* *P < 0.05*.

### Diagnosis × FHS-CVD Risk Interaction on ALFF

**Figure 1:**
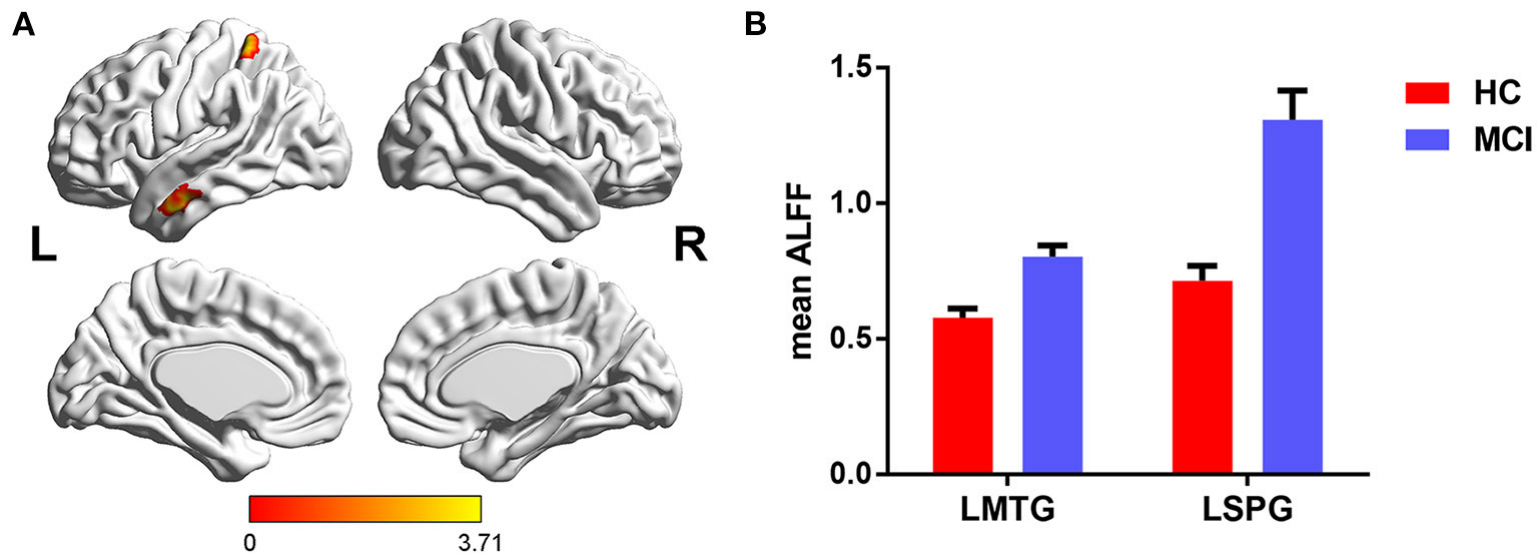
The main effects of diagnosis on ALFF and *post-hoc* analysis. **(A)** The main effects were identified in the LMTG and LSPG. **(B)** Compared with cognitively normal controls, patients with MCI exhibited increased ALFF in the LMTG and LSPG. LMTG, left middle temporal gyrus; LSPG, left superior parietal gyrus.

The main effects of diagnosis on ALFF were identified in the left middle temporal gyrus (LMTG) and left superior parietal gyrus (LSPG). Compared with cognitively normal controls, patients with MCI exhibited higher value of ALFF in the LMTG and LSPG (see Table 2 and Figure 1).

**Table 2:** Diagnosis × FHS-CVD risk interaction on ALFF.

**Brain region**	**BA**	**Peak MNI coordinates (mm)**			**Peak *F*-value**	**Cluster size (mm3)**
		**X**	**Y**	**Z**		
**Main effect of diagnosis**						
L middle temporal gyrus	21	−60	0	−24	3.59	1,134
L superior parietal gyrus	5	−33	−45	66	3.93	945
**Main effect of risk**						
L fusiform gyrus	20	−36	−9	−27	3.24	972
L precuneus	31	−2	−54	18	3.55	1,377
L cerebellum posterior lobe	/	−6	−75	−27	3.71	1,431
**Diagnosis × risk interaction**						
L hippocampus	/	−24	−6	−24	3.48	1458

*All the regions survived the multiple comparisons based on Gaussian random field theory (GRF) (voxel-wise P < 0.01, cluster-wise P < 0.05, two-tailed). L, left; BA, Brodmann's area*.

**Figure 2:**
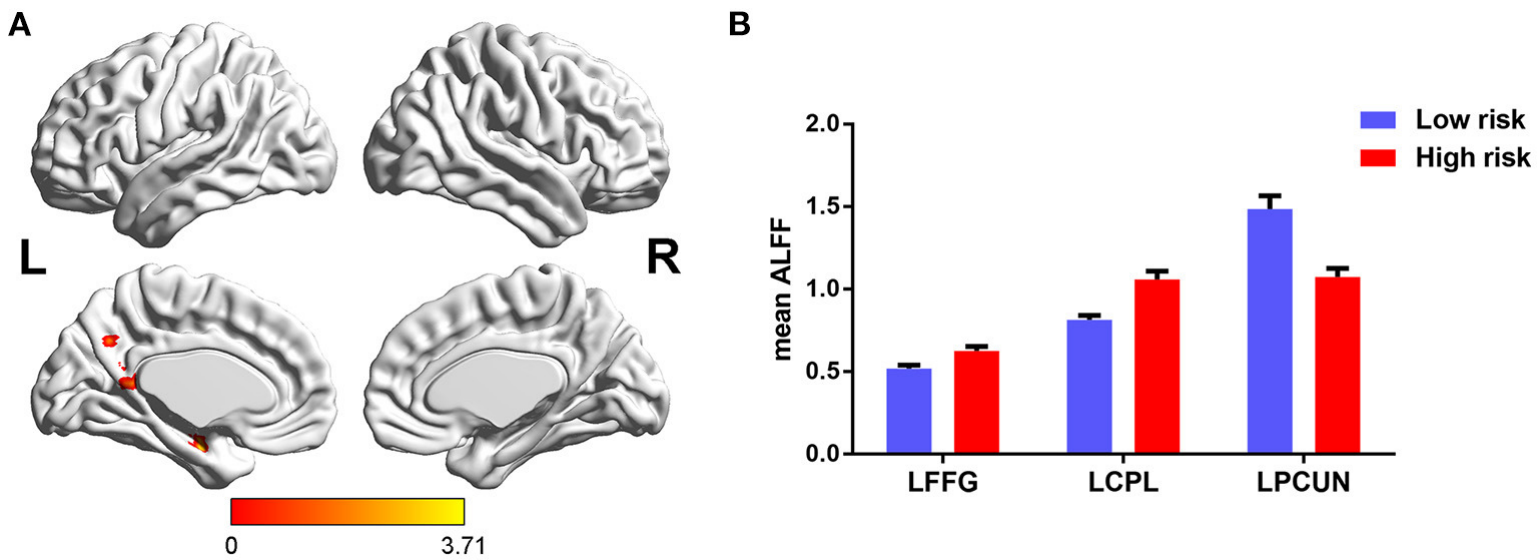
The main effects of FHS-CVD risk on ALFF and *post-hoc* analysis. **(A)** The main effects were identified in the LFFG, LPCUN, and LCPL. **(B)** Compared with subjects with low FHS-CVD risk, those with high FHS-CVD risk showed significantly increased ALFF in the LFFG and LCPL, while decreased ALFF in the LPCUN. LFFG, left fusiform gyrus; LPCUN, left precuneus; LCPL, left cerebellum posterior lobe.

The main effects of FHS-CVD risk on ALFF were observed in the left fusiform gyrus (LFFG), left precuneus (LPCUN), and left cerebellum posterior lobe (LCPL). Compared with subjects with low FHS-CVD risk, those with high FHS-CVD risk showed significantly increased ALFF in the LFFG and LCPL, while decreased ALFF in the LPCUN (see Table 2 and Figure 2).

**Figure 3:**
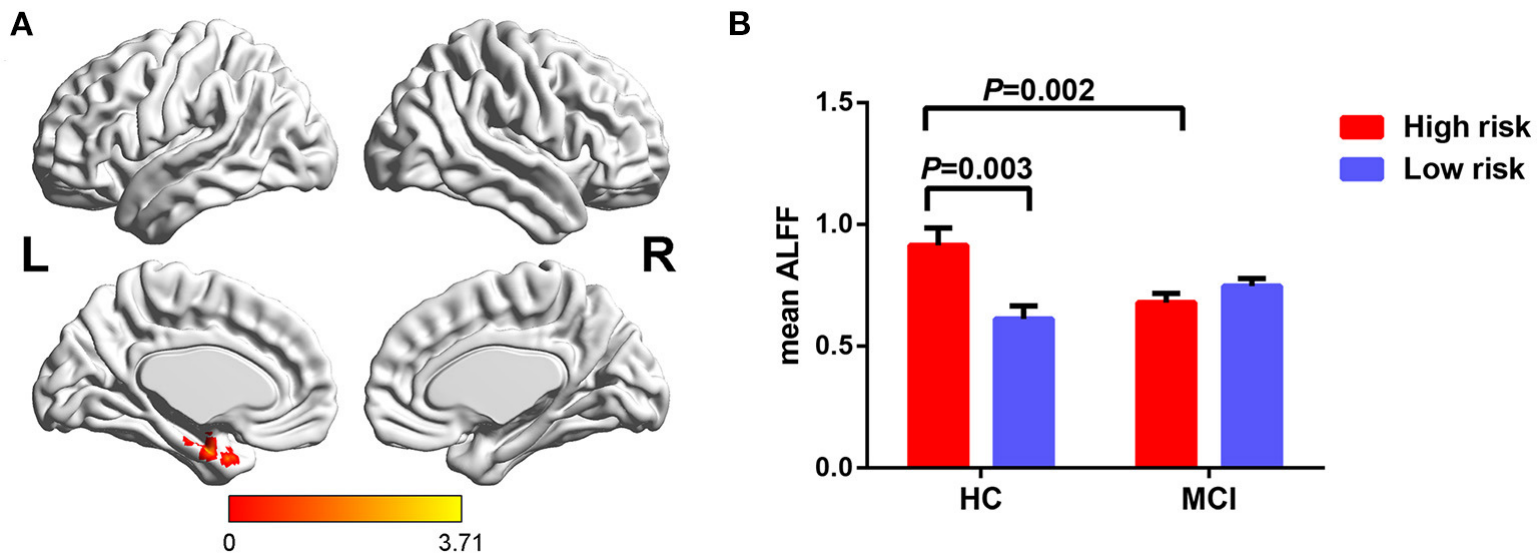
The interaction of diagnosis × FHS-CVD risk on ALFF and *post-hoc* analysis. **(A)** The interaction was seen in the LHIP. **(B)** HCs with high risk showed significantly increased ALFF in the LHIP than those with low risk, while MCI patients with high risk showed decreased ALFF in the LHIP than HCs with high risk. LHIP, left hippocampus.

Furthermore, significant interaction of diagnosis × FHS-CVD risk on ALFF was seen in the left hippocampus (LHIP). Interestingly, compared to subjects with low FHS-CVD risk, those with high risk produced opposite trajectory changes in the LHIP across HC and MCI. HCs with high vascular risk showed significantly increased ALFF in the LHIP than those with low vascular risk, while MCI patients with high vascular risk showed lower ALFF values in the LHIP than those with low vascular risk with no significance. MCI patients with high vascular risk showed decreased ALFF in the LHIP than HCs with high vascular risk (see Table 2 and Figure 3).

### Behavioral Significance

**Figure 4:**
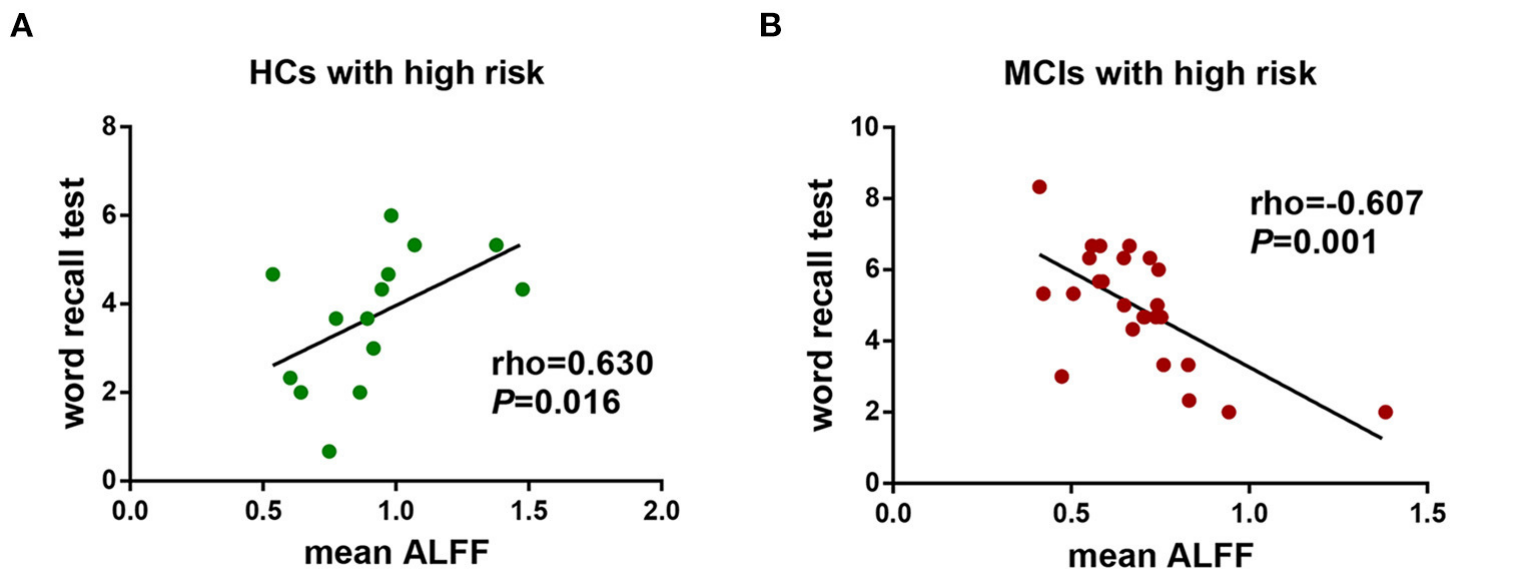
The behavioral significance of the interaction of diagnosis × FHS-CVD risk on ALFF. **(A)** ALFF in the LHIP was positively correlated with word recall test in HCs with high vascular risk (rho = 0.630, *P* = 0.016). **(B)** ALFF in the LHIP was negatively correlated with word recall test in MCI patients with high vascular risk (rho = −0.607, *P* = 0.001). LHIP, left hippocampus.

We further performed a correlation analysis to explore the behavioral significance of the interaction between diagnosis and FHS-CVD risk. We found that ALFF in the LHIP was positively correlated with word recall test in HCs with high vascular risk (rho = 0.630, *P* = 0.016), while negatively correlated with the same test in MCI patients with high vascular risk (rho = −0.607, *P* = 0.001; Figure 4).

## Discussion

In the present study, we aimed to explore whether an aggregation of vascular risk factors modulates the ALFF in patients with MCI and to identify whether it correlates with the behavioral characteristics in MCI. We found the main effects of vascular risk on ALFF were observed in the LFFG, LPCUN, and LCPL. Previous studies have demonstrated that each single vascular risk factor has an effect on the structure, function or metabolism of the brain. Subjects with hypertension showed decreased brain functional and effective connectivity (Bu et al., 2018). Hypertension was significantly associated with lower mean white matter fractional anisotropy, representing impaired integrity of the white matter microstructural (Haight et al., 2018). Many studies have demonstrated cognitive dysfunction, disruptions of functional connectivity, and white matter within the default mode network (Chen et al., 2015; Cui et al., 2015; Qi et al., 2017; Tan et al., 2019). There was significant hypoperfusion in multiple brain regions in smokers (Durazzo et al., 2015). The positive BOLD signal is known to indicate increased neuronal activity, while a negative BOLD signal indicates decreased neuronal activity, and therefor blood flow (Boorman et al., 2010). Based on this BOLD imaging principle, vascular factors themselves can cause a certain degree of abnormal signals in brain regions, and it's not surprising that the aggregation of all these vascular risk factors has an effect on the spontaneous brain activity in our present study. Further analysis showed participants with high vascular risk showed decreased ALFF in the LPCUN, while increased ALFF in the LFFG and LCPL. The predominant hypometabolism involving the precuneus was observed both in MCI and AD patients (Riederer et al., 2018). The decreased ALFF in the LPCUN in our study suggested that the precuneus is fragile to vascular damage, and participants with higher vascular risk may be in a vulnerability state for the development of neurodegenerative disorders, especially AD (Dai et al., 2008). Although the cerebellum was known to be a relatively preserved structure, studies have demonstrated the cerebellar posterior lobe contributes to higher cognitive function (Hoche et al., 2018). As the vascular risk increases, the cognitive cerebellum may be activated in compensation for deficit in the cerebral cortex (Bai et al., 2011).

Previous systematic reviews or meta-analyses of vascular risk factors for dementia and AD showed that the relative risk of diabetes, hypertension, smoking, obesity, and hyperlipemia for dementia and AD ranges from 1.24 to 3.1 (Ballard et al., 2011). As with metabolic syndrome, taking different vascular risk factors as a whole, may help to provide more comprehensive information (Frisardi et al., 2010). Few studies investigated the accumulating effect of different vascular risk factors on brain function based on resting-state fMRI. The FHS-CVD risk score is a simple and reliable tool for comprehensive assessment of the risk of cardiovascular events. In this study, we found the interaction of diagnosis and FHS-CVD risk on ALFF was in the LHIP. As the hub node of memory circuit, hippocampus plays an important role in the encoding, storage, and retrieval of memories, and it's thought to be one of the earliest brain regions to be affected in AD (Gliebus, 2018). Both fMRI and structural imaging studies have demonstrated hippocampus changes in patients with MCI, and the changes become more severe in AD patients (Leandrou et al., 2018; Xue et al., 2019). HCs with high FHS-CVD risk showed increased ALFF in the LHIP than those with low FHS-CVD risk and ALFF in the LHIP significantly positively correlated with word recall test in HCs with high FHS-CVD risk, this may represent brain function compensation in response to increased vascular risk effects. While MCI patients showed opposite trajectory changes of ALFF as the FHS-CVD risk increased and the ALFF value negatively correlated with the same test in MCI patients with high FHS-CVD risk. Compensation of early neural network connections was observed in the normal elderly and in MCI patients without vascular risk factors, while this compensation was inhibited in MCI as vascular risk factors increased (Chen et al., 2018), giving some support for our findings. Accumulating evidences highlight the significance of vascular dysfunction in AD. Neuropathological studies have found that nearly 80% of patients diagnosed with AD have vascular lesions such as microinfarcts and cerebral atherosclerosis (Toledo et al., 2013; Power et al., 2018). The animal models of AD driven by Aβ also showed microvascular morphological changes (Iadecola, 2010). Vascular risk factors, such as hypertension and diabetes may induce blood-brain barrier and neurovascular unit injury, thus causing chronic cerebral hypoperfusion to adversely affect the neuronal homeostasis and eventually lead to neuronal cell death (Goldwaser et al., 2016; Wang et al., 2018; Liesz, 2019). While individual vascular risk factors could specifically or primarily affect neurovascular unit to varying degrees, their aggregation in clusters may have a broader impact on the cerebrovascular system, including micro-vessels and macro-vessels. Vemuri et al. have demonstrated that vascular health can directly or indirectly affect neurodegeneration biomarkers, such as tau deposition in entorhinal cortex (Vemuri et al., 2017). It is identified recently that Aβ generating reactive oxygen species causes pericyte constriction of brain capillaries can lead to chronic hypoperfusion, exacerbating neurodegeneration and cognitive dysfunction in AD (Nortley et al., 2019). A clinical pathologic study found that the Aβ peptide load in MCI patients appeared intermediate between HCs and dementia due to AD (Mufson et al., 1999). In our study, one explanation for the lower ALFF of LHIP in MCI patients with high FHS-CVD risk might be that, in addition to the effects of neurodegenerative pathology, the higher FHS-CVD risk representing the greater aggregation of vascular factors may lead to more severe vascular dysfunction and reduction of cerebral blood flow in MCI, resulting in decompensation. Meta-analysis of fMRI studies has already concluded that patients with MCI showed decreased ALFF value than HCs in the LHIP (Pan et al., 2017). This kind of change may imply weakening of the activities of neurons in the hippocampus, reflecting the pathophysiological transition process of MCI to AD (Yang et al., 2018). Further longitudinal studies are needed to clarify this in the future.

Our study possessed several biological and technical limitations. First, we did not measure Aβ deposition, a major pathological biomarker of AD. There has been increasing evidence of interaction between vascular pathology and Aβ pathology, with cerebral ischemia promoting the aggregation of Aβ, which in turn further reduces cerebral blood flow (Popa-Wagner et al., 2015). Aβ deposition and the presence of cerebrovascular pathologies often co-exist in the brains of the elderly population (Attems and Jellinger, 2014; Love and Miners, 2016). Thus, although the present study demonstrated that FHS-CVD risk modulates the effect of diagnosis on cognition and cerebral function, there may be convergent pathologies that contribute to altered brain function. Second, due to the sample size of this study, further repeated studies with large sample in independent samples are needed. Finally, since it's is a cross-sectional study, we were unable to draw any causal conclusions. Longitudinal studies of the effects of FHS-CVD risk on cerebral function are still needed in the future.

In conclusion, we first assessed the vascular-imaging-behavior relationship involving the FHS-CVD risk profile in MCI patients and HCs. This study provides preliminary evidence highlighting that the aggregation of vascular risk factors modulates the spontaneous brain activity in MCI, and this may serve as a potential imaging mechanism underlying vascular contribution to AD.

## Data Availability Statement

The datasets generated for this study are available on request to the corresponding authors.

## Ethics Statement

The studies involving human participants were reviewed and approved by the Research Ethics Committee of the First Affiliated Hospital of Zhejiang University. The patients/participants provided their written informed consent to participate in this study. Written informed consent was obtained from the individual(s) for the publication of any potentially identifiable images or data included in this article.

## Author Contributions

LZ contributed to the conception and design of the work, the analysis and interpretation of data, and the manuscript writing. HN and XL helped analyze the data. JW, XL, YLin, YS, and KZ contributed to the conception, design and acquisition data. YLi contributed to the conception and design of the work. GP and BL revised the work critically. All authors reviewed and approved the submitted manuscript.

## Conflict of Interest

The authors declare that the research was conducted in the absence of any commercial or financial relationships that could be construed as a potential conflict of interest.
