# Supplementary material for: Aggregation of Vascular Risk Factors Modulates the Amplitude of Low-Frequency Fluctuation in Mild Cognitive Impairment Patients
Source: Front Aging Neurosci. 2020 Dec 21;12:604246. doi: 10.3389/fnagi.2020.604246 (PMC7779477; doi:10.3389/fnagi.2020.604246)
Supplement: Supplementary file 1 [file Table_1.docx]

Supplementary Material

# Supplementary Tables

**Table S1**. Neuropsychological profiles of MCI patients and HCs stratified by FHS-CVD risk

|  | **MCI group (N=43)** |  | **HC group (N=29)** |  | ***P*** |  |  |
| --- | --- | --- | --- | --- | --- | --- | --- |
|  | **Low (N=17)** | **High (N=26)** | **Low (N=15)** | **High (N=14)** | **diagnosis** | **risk** | **interaction** |
| MMSE | 25.00±2.87 | 26.77±1.66 | 27.13±2.33 | 28.71±0.91 | <0.001* | 0.001* | 0.851 |
| MoCA | 19.53±2.81 | 20.08±2.86 | 25.00±3.46 | 25.43±1.28 | <0.001* | 0.469 | 0.930 |
| ADAS-Cog 14 | 12.84±7.00 | 13.64±7.80 | 7.60±6.06 | 8.55±4.11 | 0.002* | 0.592 | 0.963 |
| Word recall test | 5.02±1.09 | 4.99±1.58 | 3.27±1.31 | 3.71±1.54 | <0.001* | 0.547 | 0.487 |
| Following commands | 1.24±1.15 | 1.35±0.85 | 0.75±0.77 | 0.87±0.74 | 0.027* | 0.594 | 0.977 |
| Constructions | 0.53±0.51 | 0.19±0.40 | 0.44±0.51 | 0.20±0.41 | 0.855 | 0.010* | 0.705 |
| Delayed word recall | 2.18±3.45 | 2.38±3.28 | 0.69±1.66 | 1.00±1.51 | 0.046* | 0.688 | 0.924 |
| Naming objects and fingers | 0.18±0.39 | 0.34±0.50 | 0.13±0.34 | 0.07±0.26 | 0.076 | 0.462 | 0.176 |
| Ideational praxis | 0.18±0.39 | 0.31±0.68 | 0.19±0.54 | 0.07±0.26 | 0.264 | 0.786 | 0.194 |
| Orientation | 0.65±1.11 | 0.42±0.99 | 0.13±0.50 | 0.13±0.35 | 0.060 | 0.607 | 0.575 |
| Word recognition | 1.53±1.14 | 1.46±1.39 | 0.79±0.77 | 0.98±1.25 | 0.056 | 0.761 | 0.593 |
| Recall of test instructions | 0±0 | 0.23±0.59 | 0±0 | 0±0 | 0.186 | 0.186 | 0.186 |
| Spoken language ability | 0±0 | 0.15±0.61 | 0±0 | 0±0 | 0.396 | 0.396 | 0.396 |
| Word-finding difficulty | 0.06±0.24 | 0.12±0.43 | 0±0 | 0±0 | 0.215 | 0.686 | 0.686 |
| Comprehension of spoken language | 0.24±0.44 | 0.27±0.53 | 0.06±0.25 | 0.07±0.26 | 0.077 | 0.850 | 0.887 |
| Maze test | 0.18±0.39 | 0.19±0.40 | 0.19±0.54 | 0.07±0.26 | 0.632 | 0.579 | 0.478 |
| Number cancellation | 0.88±1.32 | 1.19±1.36 | 0.69±1.14 | 1.20±1.66 | 0.934 | 0.202 | 0.719 |

Data were presented as the mean ± standard deviation (SD). P-values were obtained by two-way analysis of variance (ANOVA). *indicates a statistical difference between groups, P<0.05.
